# Modeling of Mechanical Stress Exerted by Cholesterol Crystallization on Atherosclerotic Plaques

**DOI:** 10.1371/journal.pone.0155117

**Published:** 2016-05-05

**Authors:** Yuemei Luo, Dongyao Cui, Xiaojun Yu, Si Chen, Xinyu Liu, Hongying Tang, Xianghong Wang, Linbo Liu

**Affiliations:** 1 School of Electrical and Electronic Engineering, Nanyang Technological University, Singapore, Singapore; 2 School of Chemical and Biomedical Engineering, Nanyang Technological University, Singapore, Singapore; Beijing Key Laboratory of Diabetes Prevention and Research, CHINA

## Abstract

Plaque rupture is the critical cause of cardiovascular thrombosis, but the detailed mechanisms are not fully understood. Recent studies have found abundant cholesterol crystals in ruptured plaques, and it has been proposed that the rapid expansion of cholesterol crystals in a limited space during crystallization may contribute to plaque rupture. To evaluate the effect of cholesterol crystal growth on atherosclerotic plaques, we modeled the expansion of cholesterol crystals during the crystallization process in the necrotic core and estimated the stress on the thin cap with different arrangements of cholesterol crystals. We developed a two-dimensional finite element method model of atherosclerotic plaques containing expanding cholesterol crystals and investigated the effect of the magnitude and distribution of crystallization on the peak circumferential stress born by the cap. Using micro-optical coherence tomography (μOCT), we extracted the cross-sectional geometric information of cholesterol crystals in human atherosclerotic aorta tissue *ex vivo* and applied the information to the model. The results demonstrate that (1) the peak circumference stress is proportionally dependent on the cholesterol crystal growth; (2) cholesterol crystals at the cap shoulder impose the highest peak circumference stress; and (3) spatial distributions of cholesterol crystals have a significant impact on the peak circumference stress: evenly distributed cholesterol crystals exert less peak circumferential stress on the cap than concentrated crystals.

## Introduction

The common morphometric characteristic of a vulnerable plaque is a thin fibrous cap overlying an extensive lipid-rich necrotic core [1–4]. Recent studies by Abela *et al* have indicated that cholesterol crystals may play a significant role in plaque rupture. The crystallization of cholesterol in an atheromatous plaque leads to the rapid accumulation of these crystals in a limited space. The crystals then extrude through or protrude into membranes, damaging the fibrous cap and increasing the potential of plaque rupture [5–7]. This proposal is supported by histological observations of abundant cholesterol crystals around plaque rupture sites in human pathologic specimens and *in vitro* experimental demonstrations of up to a 45% volume increase of cholesterol crystals during crystallization [5–7]. This hypothesis is also supported by a histological study by Frink, which showed the correlation between parallel cholesterol crystals and the sites of plaque rupture [8].

The physical stress on the cap can be conveniently predicted by finite element (FE) modeling [9–14]. However, cholesterol crystals, potential physical risk factors of plaque rupture, have not been included in previous mechanical models; therefore, how cholesterol crystals affect atherosclerotic plaques remains elusive. Cholesterol crystals are dissolved in tissue sections embedded in paraffin during standard histological processing and leave “clefts” in the histology sections, which may not faithfully reflect the geometry of the cholesterol crystals *in vivo*. Intravascular optical coherence tomography (IVOCT) imaging can detect cholesterol crystals *in vivo* [15–17]. However, the typical spatial resolution of IVOCT (~10–30 μm) is too coarse to accurately measure cholesterol crystals. Micro-optical coherence tomography (μOCT) is a new generation of OCT technology that can clearly characterize cholesterol crystals in intact arterial tissue *ex vivo* with 1–2 μm spatial resolution [18–20]. μOCT opens the possibility for modeling cholesterol crystallization in necrotic cores and exploring the relationship between cholesterol crystals and plaque rupture.

This study aims to investigate the stress exerted by cholesterol crystal growth through the finite element method (FEM). We modified the existing two-dimensional plaque models by adding a cholesterol crystal model based on the μOCT measurement. We chose the peak circumferential stress (PCS) as the primary risk factor in our mechanical model because a large proportion (58%) of plaques ruptures at points of PCS [10, 13, 21, 22]. Our study discovered a link between PCS and the magnitude of crystal expansion and a correlation between PCS and the spatial distribution of cholesterol crystals. The new mechanical plaque model proposed in this study improves our understanding of the physical role of cholesterol crystals in plaque rupture and creates the possibility to preventively evaluate the mechanical risk factor based on high-resolution IVOCT images acquired *in vivo*.

## Methods

### Geometry of the plaque model

**Fig 1:**
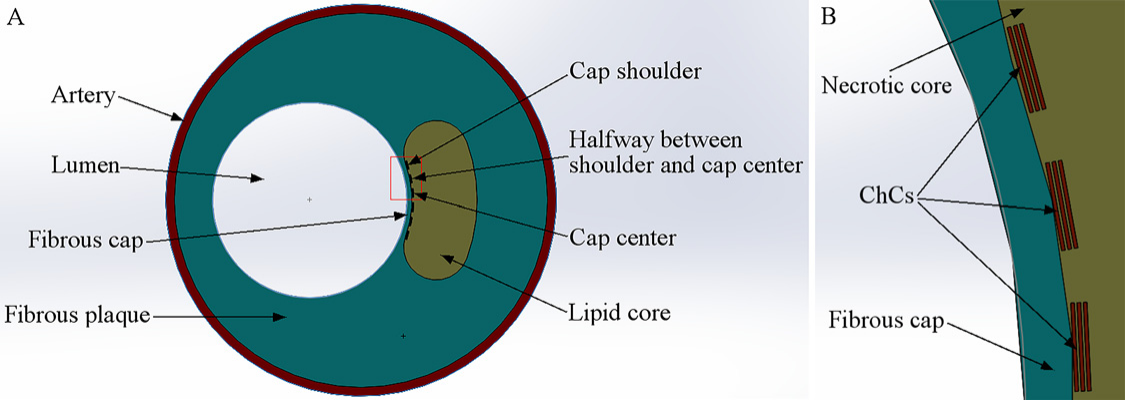
Diagram of an Idealized Cross-section of an Atherosclerotic Coronary Artery. A: Structure of the overall atherosclerotic coronary artery. B: Magnified view of the thin fibrous cap with the cholesterol crystals illustrated in the red box in A.

We propose an idealized, two-dimensional FEM of the cross-section of an atherosclerotic coronary artery based on the morphology and geometry established in previous studies [9, 12]. As illustrated in Fig 1, the lumen was modeled as a circular hole bearing the blood pressure. Cholesterol crystals are frequently observed in atheromatous plaques and are usually present in the necrotic core. The fibrous cap, the thin part between the lumen and necrotic core, is vulnerable to damage, which induces plaque rupture. During crystallization, cholesterol crystals expand in the confined space (necrotic core) and then protrude through or penetrate into the thin fibrous cap. To reproduce cholesterol crystals and their dynamics in our model, we measured the geometry of cholesterol crystals using μOCT three-dimensional volumetric imaging, followed by increasing the size of the cholesterol crystals in the depth direction.

The morphologic parameters of the artery were adopted from a previous study [12]. The mean thickness of the fibrous cap is 91 μm, with a range from 25 μm to 370 μm. The mean lumen area is 5.77 mm^2^. The arc angle and mean area of the necrotic core are 84.65 degrees and 2.22 mm^2^, respectively, with a relative mean core area of 19.17% and a relative core thickness of 49.41%. These geometrical dimensions are in close agreement with other investigations [23, 24]. In this condition, the stenosis severity is approximately 70.53%.

### Measurement of cholesterol crystal geometry using μOCT

The detailed construction of the μOCT imaging device used in this study was reported in a previous study [25]. In brief, the spatial resolution of μOCT was 1.3 μm (axial) by 2.5 μm (transverse) in air. The size of the B-scan (cross-sectional) images was 0.872 mm (width) by 1.4 mm (height). The image speed was 20 frames per second with 512 A-lines per frame. We examined formalin-fixed and non-identified aorta specimens from patients with abdominal aortic aneurysm treated by vascular surgery. The specimens were stored in 4% neutral buffered formaldehyde. We identified the regions of interest by gross visual inspection and acquired OCT images from the luminal surface of the aortas.

In the cross-sectional μOCT images, the length of cholesterol crystals was measured as the horizontal distance between the left and right boundaries of the cholesterol crystals, and the thickness was measured as the vertical distance between the top and bottom surfaces under the assumption that the refractive index was 1.33. The Institutional Review Board at Nanyang Technological University (IRB-2014-12-004) approved the studies using human arterial tissues.

### Material Properties

In the idealized model, the arterial wall and the fibrous plaque, were made of transversely isotropic materials with linear elastic properties, sharing similar mechanical properties in the circumferential (Ɵ) and axial (z) directions for each tissue. The material properties of the arterial and fibrous cap were adopted from previous studies [9, 10, 21] and are shown in Table 1: Young’s moduli E_r_, E_Ɵ_ and E_z_ (in the radial, circumferential and axial directions, respectively), Poisson ratios ν_rƟ_ and ν_Ɵz_ (in the r-Ɵ and Ɵ-z planes, respectively), and shear modulus G_rƟ_ (in the r-Ɵ plane). The fibrous cap and fibrous plaque were assumed to be continuous and shared the same material properties. The necrotic core was estimated to be a very soft (Young’s modulus E = 1 kPa) and incompressible (Poisson ratio ν = 0.49) isotropic material [9, 10, 12, 26]. Cholesterol crystals were incompressible but rigid solids; therefore, the Young’s modulus and Poisson ratio of cholesterol crystals were assumed as 100 kPa and 0.49, respectively.

**Table 1:** Material Properties of the Plaque and Artery Used in the Structural Analyses.

	E_r_ (kPa)	E_Ɵ_ = E_z_ (kPa)	ν_rƟ_	ν_Ɵz_	G_rƟ_ (kPa)
**Plaque**	50	1000	0.01	0.27	500
**Artery**	10	100	0.01	0.27	50

### Structural Analysis

The FE analysis was conducted to calculate the continual stress and strain of the fibrous cap and to investigate PCS as a result of expanded cholesterol crystals using Ansys 15.0 (Ansys, Inc., Canonsburg, Pennsylvania, USA). Due to the symmetry of this idealized model, we simulated half of the cross-section for analysis, with the nodes along the center line restricted to move in the radial (r) direction. The various regions of the artery, plaque and lipid core were meshed with 1224 eight-node quadrangular elements. The models were solved under the assumption of plane strains.

Additionally, a static pressure of 110 mmHg (14.6 kPa) acted along the lumen wall, representing the mean intracoronary blood pressure adopted from previous studies [9, 10, 26].

For simplicity, we placed only one cholesterol crystal in each location, and the effects of multiple cholesterol crystals were simulated by increasing the magnitude of expansion. This simplified model enabled us to investigate the effects of the magnitude and the spatial distributions of cholesterol crystal growth on PCS.

### Locations and loading of cholesterol crystals

We chose three representative locations at the cap: the cap shoulder, the cap center, and halfway between the shoulder and the cap center (Fig 1). Cholesterol crystal growth was implemented by expanding the cholesterol crystal in the depth (thickness) direction.

## Results

**Fig 2:**
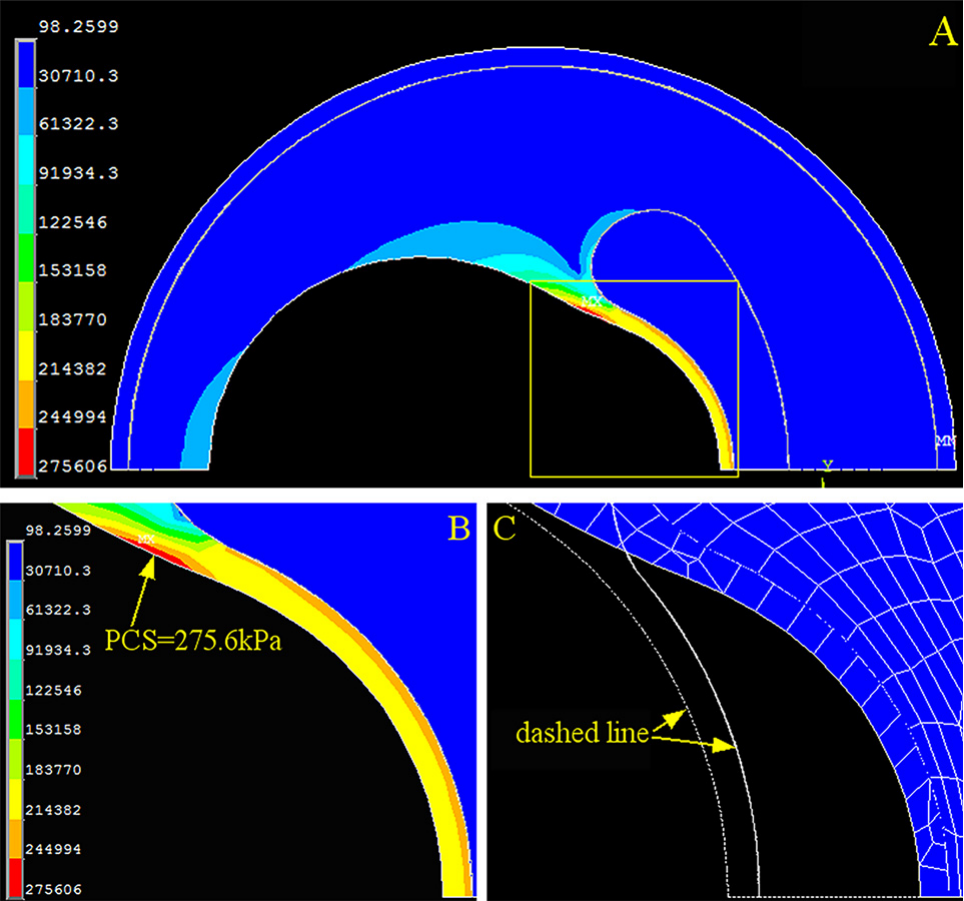
Stress and Strain of the Coronary Artery without Cholesterol Crystals. (A) Stress distribution of the overall coronary artery. (B) Contour plot of the stress on the fibrous cap (yellow dashed box in A). (C) Contour plot of the strain on the fibrous cap. The dashed line is the original contour before loading, and the meshed section is the deformed cap after loading. PCS: peak circumferential stress.

To quantitatively characterize the effects of cholesterol crystal growth on PCS, we investigated the PCS changes caused by their expansion and spatial distributions. We used the idealized model without cholesterol crystals as the reference. In this case, PCS was located at the shoulder of the cap and the entire cap was expanded toward the abluminal side by the luminal blood pressure, which agrees well with previous studies (Fig 2) [9–11]. A PCS of 275.6 kPa was the pre-loaded peak offset pressure, and deviations from this offset caused by crystal loading would indicate either stabilizing or destabilizing effects of the cholesterol crystals. We found that the loading of concentrated crystals at the cap shoulder imposed the highest risk of plaque rupture by proportionally increasing PCS, whereas the evenly distributed crystal loading along the cap mitigated this risk by exerting less PCS than concentrated crystals.

### Identification and measurement of individual cholesterol crystals using μOCT

**Fig 3:**
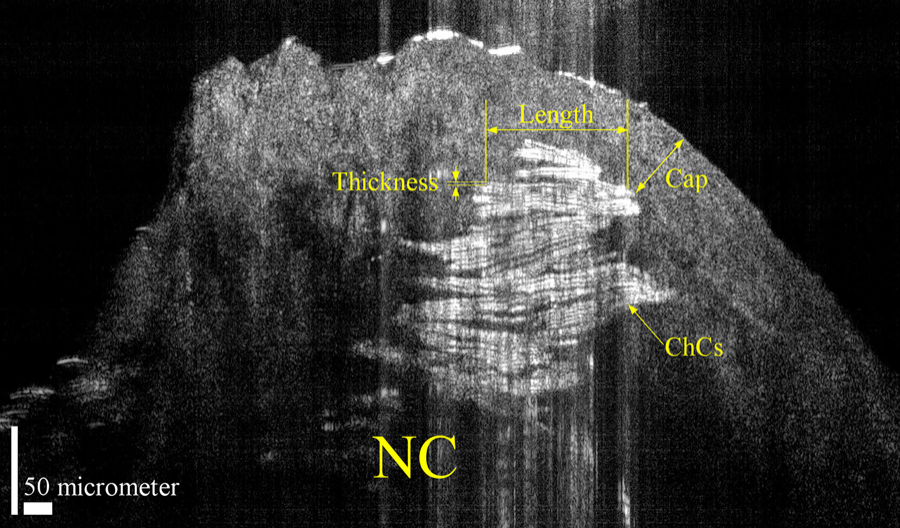
A μOCT Image of a Human Aortic Atherosclerotic Plaque Revealing the Geometries of Cholesterol Crystals. ChCs: cholesterol crystals. NC: necrotic core. Scale bar, 50 μm.

In a representative μOCT image of a human aortic atherosclerotic plaque (Fig 3), almost every cholesterol crystal in the necrotic core near the cap could be identified by its reflections from the top and bottom surface. We measured the length and thickness of 20 different cholesterol crystals from the image presented in Fig 3, and the average length and thickness were 269.1 μm and 3.0 μm, with standard deviations of 80.16 μm and 0.33 μm, respectively.

### Peak circumference stress is proportionally dependent on the cholesterol crystal growth

**Fig 4:**
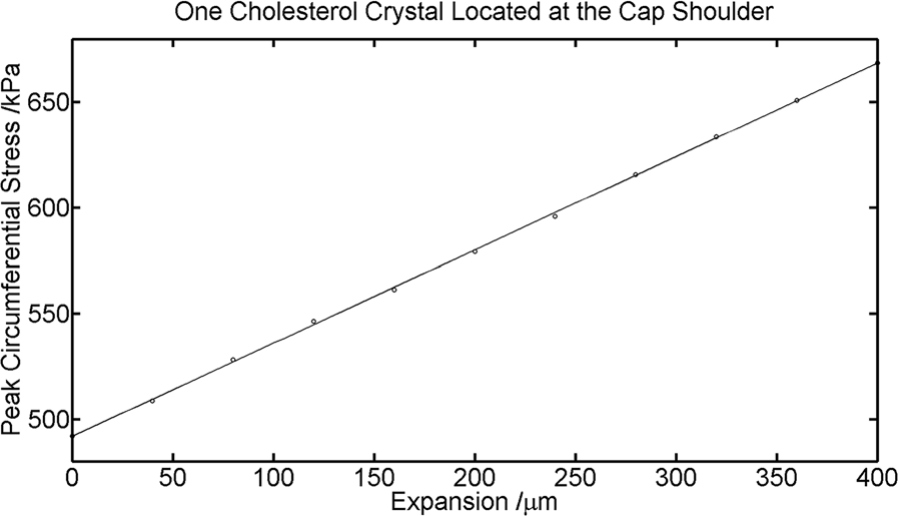
Peak Circumferential Stress (PCS) with Varying Expansion of One Cholesterol Crystal at the Cap Shoulder.

To quantitatively characterize the relationship between cholesterol crystal growth and PCS, we examined PCS during a crystal expansion of 400 μm in thickness at the cap shoulder. As expected, PCS increased as the displacement of the cholesterol crystals increased (Fig 4). PCS was almost linearly dependent on the cholesterol crystal growth within the expansion range of 0–400 μm with a slope of 0.442 kPa/μm.

### Cholesterol crystals at the cap shoulder impose the highest peak circumference stress

**Fig 5:**
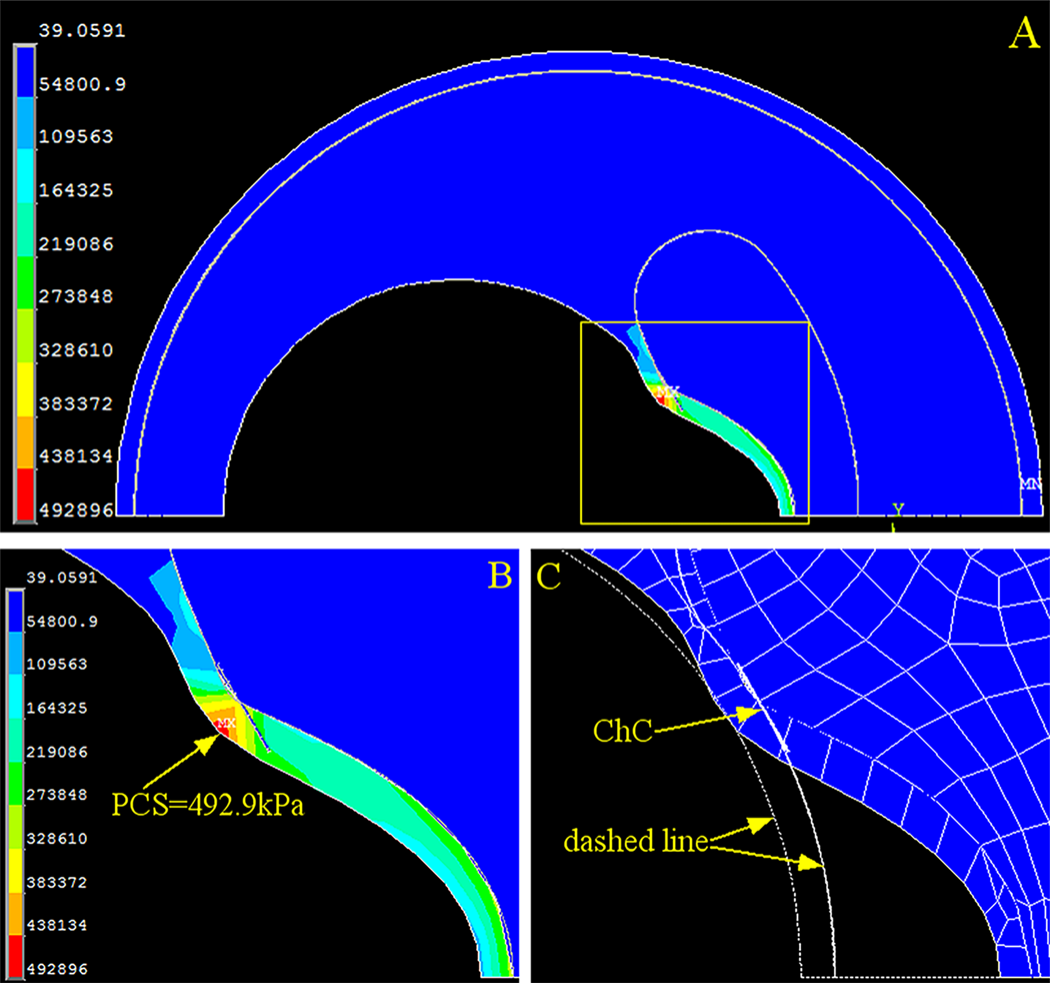
Stress and Strain of the Artery with One Cholesterol Crystal at the Cap Shoulder. (A) Stress distribution of the overall coronary artery. (B) Contour plot of the stress on the fibrous cap (yellow dashed box in A). (C) Contour plot of the strain on the fibrous cap. The dashed line is the original contour before loading, and the meshed section is the deformed cap after loading. PCS: peak circumferential stress. ChC: cholesterol crystal.

**Fig 6:**
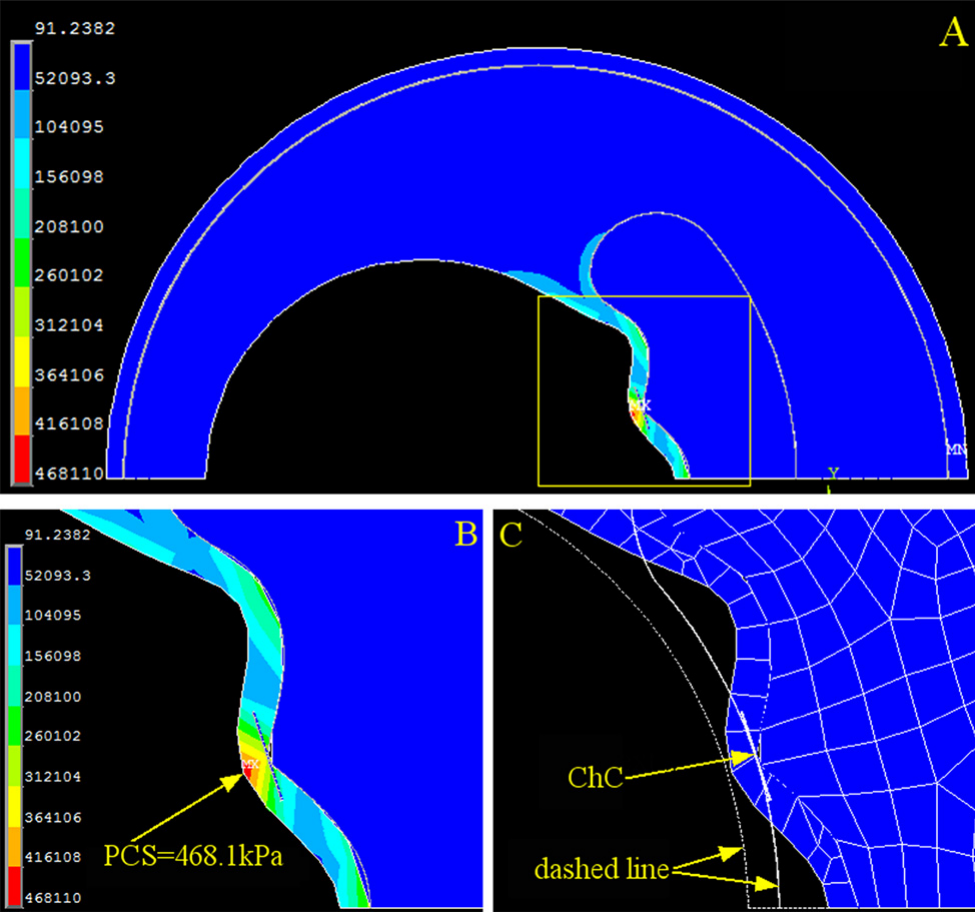
Stress and Strain of the Artery with One Cholesterol Crystal halfway between the Shoulder and the Cap Center. (A) Stress distribution of the overall coronary artery. (B) Contour plot of the stress on the fibrous cap (yellow dashed box in A). (C) Contour plot of the strain on the fibrous cap. The dashed line is the original contour before loading, and the meshed section is the deformed cap after loading. PCS: peak circumferential stress. ChC: cholesterol crystal.

**Fig 7:**
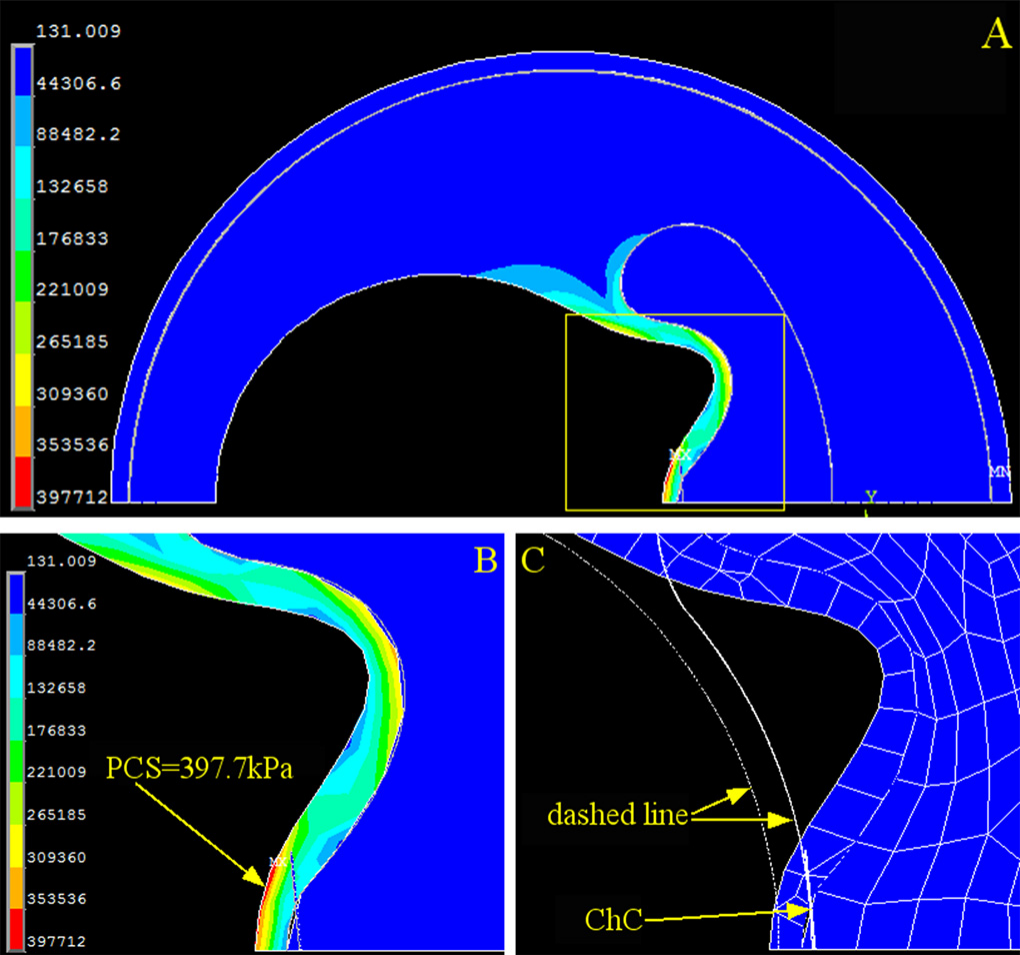
Stress and Strain of the Coronary Artery with One Cholesterol Crystal at the Cap Center. (A) Stress distribution of the overall coronary artery. (B) Contour plot of the stress on the fibrous cap (yellow dashed box in A). (C) Contour plot of the strain on the fibrous cap. The dashed line is the original contour before loading, and the meshed section is the deformed cap after loading. PCS: peak circumferential stress. ChC: cholesterol crystal.

We also investigated PCS changes caused by cholesterol crystal growth at three different locations: the shoulder (Fig 5), halfway between the shoulder and the cap center (Fig 6), and the cap center (Fig 7). At each location, cholesterol crystal growth of 2 μm caused a significant increase in PCS: 78.85% at the shoulder (Fig 5), 69.85% halfway between the shoulder and the cap center (Fig 6), and 44.30% at the cap center (Fig 7). The locations of the PCS coincided with the positions of the cholesterol crystal growth, indicating that the crystal growth was the primary risk factor in this idealized model. The crystal growth also caused significant cap deformation toward the lumen center at the shoulder, halfway between the shoulder and the cap center, and the cap center.

### Effect of the spatial distribution of cholesterol crystals on the peak circumference stress

**Fig 8:**
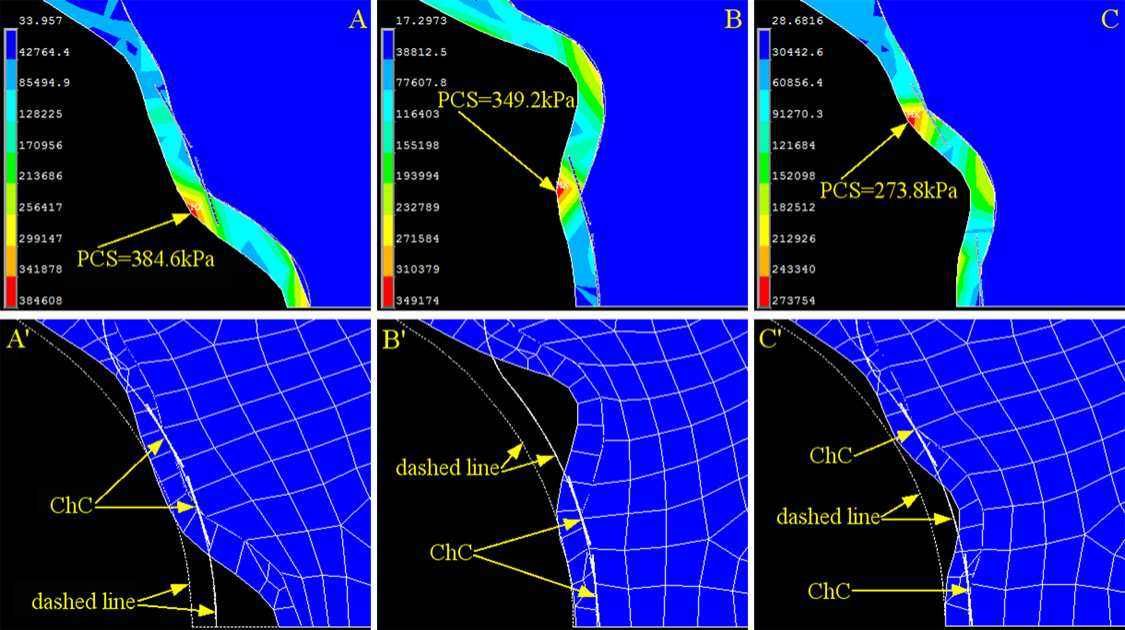
Stress and Strain of the Cap with Two Cholesterol Crystals at any Two Random Positions. (A) Contour plot of the stress on the fibrous cap when cholesterol crystals are located at the shoulder and halfway between the shoulder and the cap center. (A’) Corresponding strain contour of the cap of A. (B) Contour plot of the stress on the fibrous cap when cholesterol crystals are located halfway between the shoulder and the cap center and at the cap center. (B’) Corresponding strain contour of the fibrous cap of B. (C) Contour plot of the stress on the fibrous cap when cholesterol crystals are located at the shoulder and at the cap center. (C’) Corresponding strain contour of the fibrous cap of C. The dashed line is the original contour before loading, and the meshed section is the deformed cap after loading. PCS: peak circumferential stress. ChC: cholesterol crystal.

There are typically multiple expanding cholesterol crystals distributed along the cap. To understand the effect of the spatial distribution of cholesterol crystals on PCS, we conducted simulations to investigate the simplest case: two cholesterol crystals located in any two of the three above-mentioned cap positions. The results summarized in Fig 8 show that the distributed cholesterol crystal loading may increase PCS (384.6 kPa in Fig 8A and 349.2 kPa in Fig 8B) but to a lesser extent than in the cases of concentrated loading (Figs 5, 6 and 7) or may even maintain the PCS (273.8 kPa; Fig 8C) compared with the control case (275.6 kPa; Fig 2). The corresponding maximum cap deformation (Fig 8A’, 8B’ and 8C’) was also significantly smaller than those of the concentrated loading cases.

**Fig 9:**
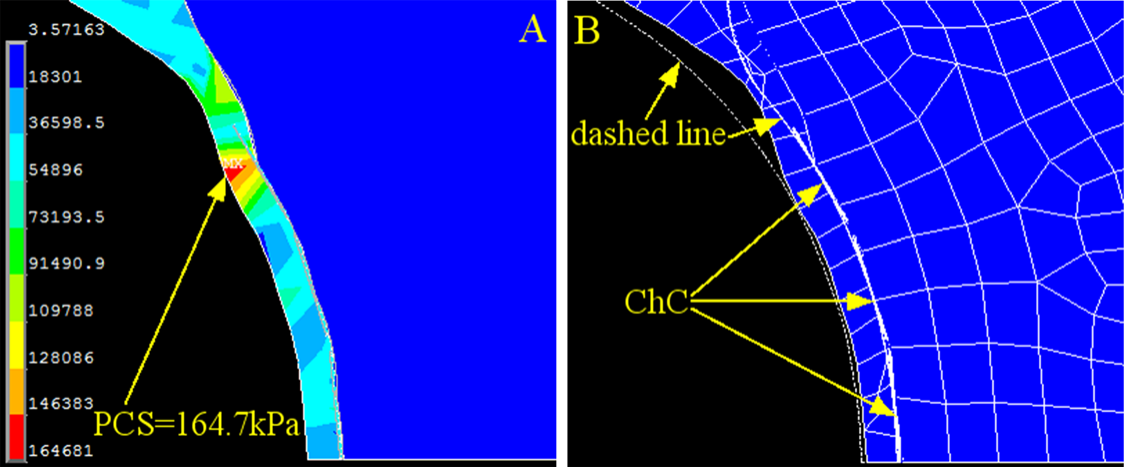
Stress and Strain of the Cap with Three Cholesterol Crystals at Three Positions. (A) Contour plot of the stress on the fibrous cap. (B) Corresponding strain contour of the fibrous cap in A. The dashed line is the original contour before loading (control case), and the meshed section is the deformed cap after loading. PCS: peak circumferential stress. ChC: cholesterol crystal.

The results of three cholesterol crystals distributed at three locations confirmed the findings in the case of two cholesterol crystals (Fig 9A), but the PCS was even smaller (164.7 kPa) and was collocated with the cholesterol crystal near the shoulder of the cap. Because the three cholesterol crystals were distributed more uniformly than the case of two cholesterol crystals, the strain of the cap (Fig 9B) was smaller and was almost the same as in the control case. These results indicate that the spatial distribution of the cholesterol crystal loading may alter the magnitude and location of PCS: evenly distributed cholesterol crystal loading may balance the stress caused by blood pressure and reduce the PCS magnitude.

### Sensitivity analysis

The sensitivity analysis of PCS on the cap was performed for all models by changing the Young’s modulus of cholesterol crystals from -50% to 1000%. Under consideration of such variations, the value of the PCS on the cap altered within 2.16%, demonstrating reasonable errors in the Young’s modulus of cholesterol crystals with minimal influence on the conclusions of this study.

## Discussion

Cholesterol crystals have long been detected in histology images as clefts in the necrotic core around the cap [4]. Abela *et al* proposed that cholesterol crystals physically penetrate into the fibrous cap, increasing the likelihood of plaque rupture [5–7]. However, due to the technical challenge of detecting individual crystals in the native tissue state, this potential risk factor was not investigated in previous coronary atherosclerotic models [9–14].

In this study, we used μOCT imaging to obtain high-resolution measurements of individual cholesterol crystals in intact human arterial tissue. With the geometric information obtained by μOCT, the physical risk factor of cholesterol crystals was investigated, for the first time, in an idealized plaque model. The results of this work provide new insight into the role of cholesterol crystals in the physical mechanism of plaque rupture.

We found that PCS collocated with the cholesterol crystals when they were concentrated in one location of the cap in the idealized plaque model (Figs 5, 6 and 7), and PCS was proportionally dependent on the cholesterol crystal expansion, placing the vessel wall at a higher risk of plaque rupture. Moreover, cholesterol crystal growth compromises cap stability by causing cap deformation (Figs 5, 6 and 7). Previous studies predicted that PCS often occurs on the shoulder of the cap, whereas the histological study by Maehara *et al* found that 63% of plaque ruptures 254 patients were on the shoulder, and the other 37% were on the center of the cap [27]. Our results indicate that PCS occurs at positions other than the shoulder with the presence of cholesterol crystals, which agrees with the histological study by Maehara *et al*.

This research revealed that the magnitude of PCS changes significantly depending on the distribution of the cholesterol crystals. When all cholesterol crystals were concentrated at one position (Figs 5, 6 and 7), PCS was significantly higher than in the cholesterol crystal-free model (Fig 2) because the region of the cap in contact with the concentrated cholesterol crystals bears the combined stress of luminal blood and cholesterol crystal expansion, whereas the rest of the cap is only under luminal blood pressure.

The situation was different when the cholesterol crystals were distributed at multiple locations rather than concentrated at one location on the abluminal side of the cap. Evenly distributed cholesterol crystals with an expansion of 2 μm acted against the stress loaded by the blood pressure and consequently reduced PCS with regards to the concentrated cholesterol crystals or to an even lower PCS than in the cholesterol crystal-free control case. This finding indicates, as far as mechanical risk factors are concerned, that the plaque was less vulnerable to rupture because as the cholesterol crystals continued to expand, the stress exerted by the cholesterol crystals eventually counteracted the blood pressure to the extent that PCS was lower than the cholesterol crystal-free control.

### Effect of nonlinearity of material properties

**Fig 10:**
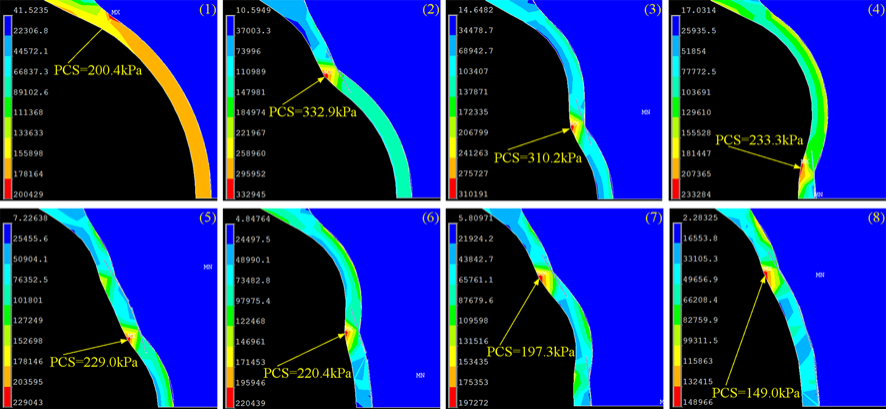
Summary of the Cap Stress in Eight Idealized Models with Non-linear Solutions. (1) Cap stress without cholesterol crystals. (2) Cap stress with one cholesterol crystal at the cap shoulder. (3) Cap stress with one cholesterol crystal halfway between the shoulder and cap center. (4) Cap stress with one cholesterol crystal at the cap center. (5) Cap stress with cholesterol crystals located at the shoulder and halfway between the shoulder and cap center. (6) Cap stress with cholesterol crystals located halfway between the shoulder and cap center and at the cap center. (7) Cap stress with two cholesterol crystals at the cap shoulder and at the cap center. (8) Cap stress with three cholesterol crystals located at three positions.

To test whether the linear solution assumed by our current study may be a good approximation of the biomechanical system under investigation, we conducted another analysis by applying the non-linear solutions into the all idealized models of artery without and with cholesterol crystals, in which the artery, plaque and necrotic core were assumed as Mooney-Rivlin materials with non-linear properties [28]. We found that the results (Fig 10) are agreed with those obtained using the linear solution well, except that PCS values resulted from the nonlinear properties were reduced compared to those obtained using the linear properties. Therefore, assumption of non-linear material properties (artery, plaque and necrotic core) does not change the conclusion that cholesterol crystals at the cap shoulder impose the highest PCS. This result suggests that, as far as the current study is concerned, the linear solution may be enough to characterize the effect of cholesterol crystallization on PCS and the mechanical risk factor of cholesterol crystals.

### Response of the peak circumferential stress with respect to intracoronary blood pressure

**Fig 11:**
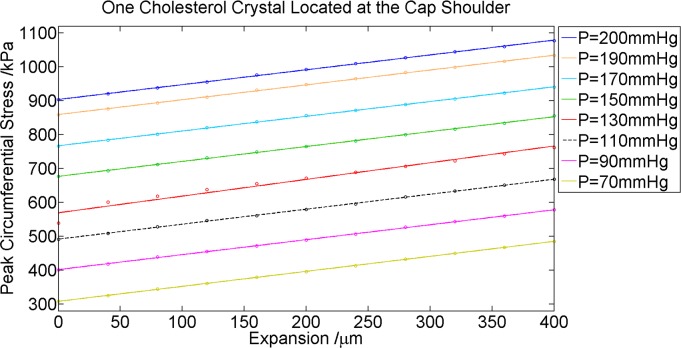
Effect of crystal expansion at the Cap Shoulder on Peak Circumferential Stress with Varying Blood Pressure. *P*: intracoronary blood pressure.

To investigate whether intracoronary pressure plays a role in the effect of cholesterol crystal expansion on PCS, we varied the lumen pressure *P* from 70 mmHg (9.3 kPa) to 200 mmHg (26.6 kPa). We found that the PCS location corresponded to the location of the concentrated cholesterol crystal regardless of luminal blood pressure change. The results (Fig 11) also show that, although intracoronary blood pressure increase elevates the baseline PCS (when crystal expansion = 0), it did not have any effect on the linear relationship between crystal expansion and PCS (Fig 11).

### Study limitation

The current study was conducted using a previously reported idealized cross-section of the coronary artery [9]. The mechanical and/or structural differences from the actual situation may result in prediction errors. In addition to tangentially arranged cholesterol crystals, randomly oriented cholesterol crystals were observed. In the current study, we developed a simple model to investigate the effect of tangentially arranged cholesterol crystal growth on PCS without covering the effect of crystal orientation.

## Conclusions

This study investigated the effects of the magnitude and locations of cholesterol crystal growth on PCS in an idealized plaque model, which is related to plaque rupture of the coronary artery. Cholesterol crystal growth concentrated at one location of the fibrous cap increases the risk of plaque rupture; we demonstrated that the magnitude of PCS was collocated with and proportional to the expansion of cholesterol crystals. In particular, the shoulder region of the fibrous cap is more susceptible to erosion/rupture induced by cholesterol crystal growth than other locations; the closer the cholesterol crystals are from the shoulder, the higher the PCS. Additionally, the spatial distribution of the stress plays a significant role in the overall effect of the cholesterol crystal expansion on PCS: the evenly distributed cholesterol crystals exert less PCS on the cap than the concentrated crystals. The simulation model developed in this research allows us to preventively evaluate the plaque vulnerability induced by cholesterol crystal growth based on high-resolution images acquired using intravascular OCT in humans.
